# The gut-placenta axis in preeclampsia: unraveling the regulatory network and clinical prospects in pathogenesis

**DOI:** 10.3389/fcimb.2025.1697739

**Published:** 2025-12-23

**Authors:** Ningxia Ma, Haibo Cao, Yao Ma, Jiaojiao Yin, Jing Yan, Jianying Pei, Chong Zhang

**Affiliations:** 1 Department of Clinical Laboratory Center, Gansu Province Maternity and Child-care Hospital, Lanzhou, Gansu, China; 2 Department of Ultrasound Medical Center, Gansu Provincial Maternity and Child-care Hospital, Lanzhou, Gansu, China

**Keywords:** preeclampsia, gut microbiota, placental exosomes, maternal-fetal interface immune microenvironment, vascular dysfunction

## Abstract

Preeclampsia (PE) is a pregnancy-specific hypertensive disorder that develops after 20 weeks of gestation, characterized by hypertension and proteinuria or multi-organ dysfunction. representing a leading cause of maternal and perinatal morbidity and mortality worldwide. The pathogenesis of PE is complex and remains incompletely understood, involving shallow placentation, endothelial dysfunction, immune imbalance, and systemic inflammation;however, the initiating triggers remain are unclear. Recent research has highlighted the gut microbiota-often termed the “second genome” -for its critical role in metabolic and immune homeostasis. Dynamic alterations in maternal gut microbial composition during pregnancy are closely associated with maternal and fetal health. Concurrently, placental exosomes, have emerged as key mediators of intercellular communication. These membrane-bound extracellular vesicles, released by placental cells, are capable of delivering microRNAs, proteins, and lipids into the maternal circulation to exert systemic effects. The emerging concept of a “gut microbiota-placental -exosome axis” suggests a pivotal role in PE progression. This review explores the bidirectional interactions between gut microbiota and placental exosomes, their regulatory impact on maternal-fetal immune crosstalk and endothelial function, and their contribution to PE pathophysiology. We also identify current research gaps and propose future directions, offering a theoretical basis for early biomarkers and targeted therapies for PE.

## Graphical Abstract

**Figure:**
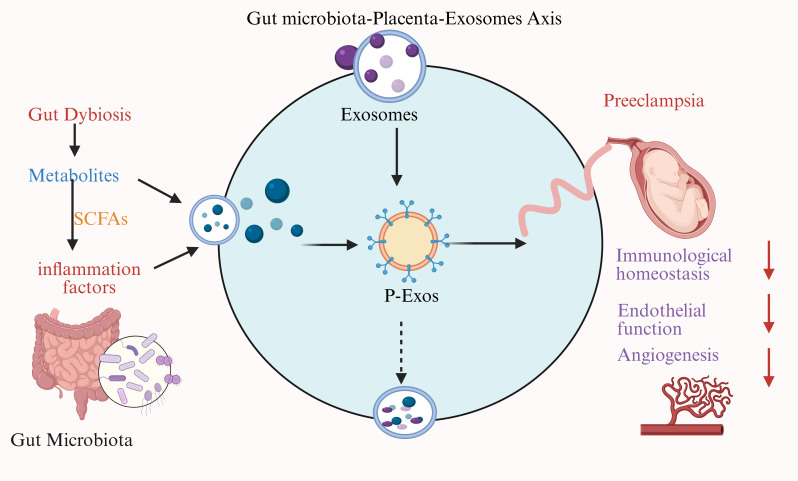
The gut-placenta axis in preeclampsia pathogenesis. This schematic illustrates the bidirectional crosstalk between maternal gut microbiota and placental exosomes in PE development. Gut dysbiosis leads to altered metabolite production (decreased SCFAs, increased LPS) and systemic inflammation. Concurrently, the stressed placenta releases exosomes containing pathogenic microRNAs and anti-angiogenic factors (sFlt-1, sEng). These two pathways converge to promote endothelial dysfunction and immune dysregulation at the maternal-fetal interface, ultimately manifesting as preeclampsia. Red arrows indicate pathogenic pathways; blue indicates protective mechanisms disrupted in PE. This framework represents a novel integration of microbiome research with placental biology.

## 1 Introduction

PE is one of the most serious hypertensive disorders of pregnancy, posing a significant global threat to both maternal and fetal health, with an estimated incidence ranging from 2% to 8% worldwide. Beyond its acute complications, such as fetal growth restriction, preterm birth, and stillbirth.PE is increasingly recognized for its long-term impact on maternal health, including an elevated risk of cardiovascular disease, chronic hypertension, and type 2 diabetes (Korzeniewski et al., 2022; Kokori et al., 2024; Marciano et al., 2025). The complexity of this multifaceted disorder has prompted ongoing efforts to elucidate its underlying pathogenesis, with growing interest in the interplay between gut microbiota and placenta-derived exosomes as potential contributors to its development and progression.

The classical model of PE pathogenesis is generally described as a two-stage process. The initial stage involves in complete trophoblast invasion and impaired remodeling of maternal spiral arteries, resulting in placental hypoperfusion, ischemia, and hypoxia (Roberts and Gammill, 2005; Jacobsen et al., 2023). During the second stage, the hypoxic placenta secretes various harmful factors into the maternal circulation, including anti-angiogenic factors such as soluble fms-like tyrosine kinase-1 (sFlt-1) and soluble endoglin (sEng), as well as pro-inflammatory cytokines. These factors collectively contribute to systemic endothelial dysfunction and multi-organ injury (Rana et al., 2022; Shi et al., 2024). However, this two-stage paradigm does not fully account for the clinical heterogeneity observed in PE. Critical questions remain unanswered: Why do some pregnant women develop shallow trophoblast invasion? What triggers the placenta to release these pathogenic factors? These unresolved issues suggest that more complex upstream initiators may precede placental dysfunction, pointing to a multi-factorial pathophysiology that extends beyond the placenta itself.

Recent systematic reviews have comprehensively characterized the maternal gut microbiota’s dynamic changes during pregnancy and their implications for maternal-fetal health (Colonetti et al., 2023; Koren and Konnikova, 2024; Torres-Torres et al., 2025). During a normal pregnancy, the maternal gut microbiota experiences adaptive changes in composition and function to meet heightened metabolic demands and support fetal development (Ahmadian et al., 2020). Nevertheless, when this dynamic equilibrium is disturbed—leading to gut dysbiosis—it has increasingly been linked to various pregnancy-related complications, such as gestational diabetes mellitus (GDM), maternal obesity and PE (Colonetti et al., 2023; Jordan et al., 2024).

In recent years, advances in research on the human symbiotic microbiome have profoundly reshaped our understanding of health and disease. Among these microbial ecosystems, the gut microbiota represents the largest and most complex community, playing a pivotal role in host nutrient metabolism, immune system maturation, and defense against pathogenic invasion (Tian et al., 2024). During a normal pregnancy, the maternal gut microbiota experiences adaptive changes in composition and function to meet heightened metabolic demands and support fetal development (Sinha et al., 2023). Nevertheless, when this dynamic equilibrium is disturbed-leading to gut dysbiosis, it has increasingly been linked to various pregnancy-related complications, such as gestational diabetes mellitus (GDM), maternal obesity and PE (Sajdel-Sulkowska, 2023; Giugliano et al., 2025).

Furthermore, extracellular vesicles (EVs), especially exosomes, are increasingly acknowledged as crucial mediators of intercellular communication (Nazimek et al., 2015; Théry and Louvard, 2025). Exosomes are nano-sized vesicles that are released into the extracellular environment through the fusion of multi-vesicular bodies with the plasma membrane (Wang et al., 2023). They are packed with a variety of bioactive molecules from their parent cells, including nucleic acids (such as miRNAs, lncRNAs, and circRNAs), proteins, and lipids (Kalluri and LeBleu, 2020).

During pregnancy, the syncytiotrophoblasts of the placenta continuously secrete large quantities of exosomes into the maternal circulation. These placenta-derived exosomes, known as P-Exos, serve as key messengers in maternal-fetal communication (Sarker et al., 2014; Tong and Chamley, 2015). Upon reaching distal maternal target cells-such as endothelial and immune cells, P-Exos can be actively internalized, thereby influencing vascular function and immune regulation to accommodate the physiological demands of pregnancy (Bai et al., 2021; Rosenfeld, 2024). While the individual roles of gut microbiota and placental exosomes in PE have been extensively explored, their potential interconnection remains largely under-investigated. This presents a critical knowledge gap in understanding the upstream regulatory networks and downstream biological consequences that might link the maternal gut to placental health and disease.

Given the important roles of the gut microbiota and exosomes in PE, it is vital to elucidate their alterations in PE and their specific effects on its pathogenesis, which may shed light on PE diagnosis and treatment. Furthermore, there exists an intimate interplay between exosomes and the gut microbiota. It is also worthwhile to investigate whether this interaction affects the progression of PE.

## 2 Role of gut microbiota in preeclampsia

Accumulating clinical and animal studies have demonstrated that the occurrence of PE is closely associated with significant alterations in the maternal gut microbiota (**Table 1**). This dysbiosis manifests in two key aspects: abnormal changes in microbial community composition and, more importantly, functional disorders. These alterations ultimately lead to the production of harmful metabolites and the initiation of systemic inflammation (Li et al., 2022; Zong et al., 2023; Torres-Torres et al., 2025).

**Table 1:** Summary of gut microbiota alterations and their clinical relevance in PE model.

Study subjects/Sample	Methodology	Major microbiota alterations	Clinical relevance/Conclusions	References
PE pregnant rat model (n=12/group)	RUPP rat model + 16S rRNA sequencing + FMT	FMT from PE donors: *Proteobacteria*↑, *Lactobacillus*↓	Demonstrated that gut dysbiosis from PE patients can directly induce PE-like symptoms (hypertension, proteinuria, placental pathological changes)	(Lv et al., 2019)
Gestational hypertensive rat model (n=10/group)	L-NAME-induced PE model + Metagenomic sequencing + Metabolomics	LPS-producing bacteria (*Escherichia*, *Enterobacter*)↑ SCFA-producing bacteria ↓	LPS-TLR4 pathway activation leads to placental inflammation and vascular endothelial dysfunction	(Chen et al., 2020)
*In vitro* placental trophoblasts + Bacterial metabolite treatment	HTR-8/SVneo cell line + Bacterial culture supernatant treatment	*Klebsiella pneumoniae* culture supernatant rich in endotoxins	Pathogenic bacterial metabolites directly impair trophoblast invasive capacity, affecting placental vascular remodeling	(Wang et al., 2019)
Germ-free mice + FMT from PE patients (n=8/group)	Germ-free C57BL/6 mice + Human fecal microbiota transplantation + Pregnancy induction	Mice receiving PE microbiota: Firmicutes/Bacteroidetes ratio ↑ *Akkermansia* ↓	Mice transplanted with PE microbiota developed glucose intolerance, insulin resistance, and hypertension.	(Crusell et al., 2018)
*In vitro* vascular endothelial cells + Bacterial co-culture	HUVEC cells + Enterobacteria isolated from PE patients	High abundance of Enterobacteriaceae (E. coli, Klebsiella)	Pathogenic bacteria directly activate the endothelial NF-κB pathway, increase inflammatory cytokines release, and impair vascular function.	(Amarasekara et al., 2015)
Gestational diabetes-PE comorbidity mouse model (n=12/group)	High-fat diet + STZ induction + 16S sequencing	*Desulfovibrio*, Bilophilab Faecalibacterium, *Roseburia*↓	Increased sulfate-reducing bacteria produce H_2_S, exacerbating placental oxidative stress and vasoconstriction	(Hu et al., 2019)
*In vitro*: Effect of microbial SCFAs on placental function	Human placental extravillous trophoblasts + Butyrate, propionate treatment	Simulation of healthy pregnant women (high SCFA) vs PE patients (low SCFA) environment	SCFA deficiency leads to increased trophoblast apoptosis and decreased angiogenic factor expression	(Gomez-Arango et al., 2016)
Inflammatory bowel disease-PE susceptibility mouse model (n=10/group)	DSS-induced colitis + Pregnancy + Microbiota analysis	Opportunistic pathogens (Citrobacter, Proteus)↑significant decrease in microbial diversity	Intestinal inflammation affects maternal immunity through the gut-liver-placenta axis, increasing PE development risk	(Jie et al., 2017)
27PE+13SP+14 Control=27NOR	Illumina HiSeq sequencing platform was used to sequence the 16S rRNA	Alpha diversity↑ Bacteroidetes/Firmicutes/Proteobacteria↑	significant changes in intestinal flora between preeclampsia patients and healthy controls	(Li G. et al., 2025)

FMT, Fecal microbiota transplantation.

### 2.1 Characteristics of gut dysbiosis in patients with PE

#### 2.1.1 Gut microbiota alterations in preeclampsia: compositional changes and metabolite imbalance

Compared to healthy pregnant women, PE patients exhibit significantly reduced gut microbial alpha-diversity, as confirmed by a recent meta-analysis of 6 studies (n=479) showing lower Shannon index (SMD: -0.47; 95% CI: -0.77 to -0.18; P = 0.02) (Colonetti et al., 2023). A comprehensive scoping review of 23 studies identified consistent patterns: preeclampsia is associated with gut dysbiosis characterized by deficient *Akkermansia, Bifidobacterium*, and *Coprococcus* but enriched *Campylobacterota* (Jordan et al., 2024). as detailed in **Table 2**. Beyond compositional shifts, the gut microbiota plays a vital role in host metabolism, with alterations in its metabolite profile significantly impacting systemic physiological functions.

**Table 2:** Key gut microbiota alterations in preeclampsia (Jordan et al., 2024).

Bacterial group	Change in PE	Functional impact	References
Lactobacillus	Decreased	Reduced SCFA production, weakened gut barrier	(Fusco and Lorenzo, 2023; Mukhopadhya and Louis, 2025)
Bifidobacterium	Decreased	Impaired immune homeostasis, increased inflammation	(Gavzy et al., 2023; Segui-Perez et al., 2025)
Proteobacteria	Increased	Enhanced LPS release, systemic inflammation	(Zhang et al., 2024; Kroon et al., 2025)
Bacteroides fragilis	Decreased	Reduced regulatory T cell induction	(Zhou et al., 2020; Zhang et al., 2022)
Firmicutes/Bacteroidetes ratio	Increased	Metabolic dysregulation, altered energy harvest	(Nkosi et al., 2022; Tsai et al., 2025)
Streptococcus	Variable	Depends on species; some pro-inflammatory	(Mitchell and Mitchell, 2010; Narciso et al., 2025)

An increasing body of evidence suggests that imbalances in gut microbial metabolism, particularly dysregulation of SCFA and TMAO pathways, contribute to the immune dysregulation and endothelial dysfunction characteristic of PE (Liu et al., 2025). SCFAs, primarily composed of acetic acid, propionic acid, and butyric acid, are produced through a key metabolic pathway whereby gut bacteria ferment dietary fiber (Cui et al., 2023). These compounds serve not only as primary energy sources for intestinal epithelial cells but also exhibit broad regulatory functions throughout the body. Specifically, butyrate inhibits histone deacetylases (HDACs), facilitating epigenetic regulation while promoting the differentiation of regulatory T cells (Tregs) and suppressing inflammatory responses (Sivaprakasam et al., 2016). Acetic acid and propionic acid can regulate blood pressure and sympathetic nervous system activity through G protein-coupled receptors (such as GPR41 and GPR43) (Maruta and Yamashita, 2020). The reduction in SCFA levels in the stool and serum of PE patients suggests that their anti-inflammatory, immunomodulatory, and vascular protective effects are diminished, which could result in immune imbalance and endothelial dysfunction (Chen et al., 2022). The TMAO pathway produces a metabolite generated when gut bacteria metabolize dietary precursors such as choline and L-carnitine, converting them first into trimethylamine (TMA), which is then absorbed into the liver via the portal vein where it is oxidized to TMAO by flavin monooxygenase 3 (FMO3). Elevated TMAO levels are strongly associated with increased cardiovascular disease risk, as TMAO promotes platelet hyper-reactivity, foam cell formation, and inhibits reverse cholesterol transport (Ussher et al., 2013). Recent studies have found that serum TMAO levels in PE patients were significantly elevated and positively correlated with disease severity (Wen et al., 2019). During pregnancy, elevated TMAO may affect placental angiogenesis and function, thereby impacting fetal growth and development (Wang et al., 2024). Therefore, increased TMAO generation driven by specific microbial alterations may represent a key molecular link between gut dysbiosis and PE vascular lesions.

### 2.2 Pathogenic mechanisms linking gut microbiota dysbiosis to preeclampsia

**Figure 1:**
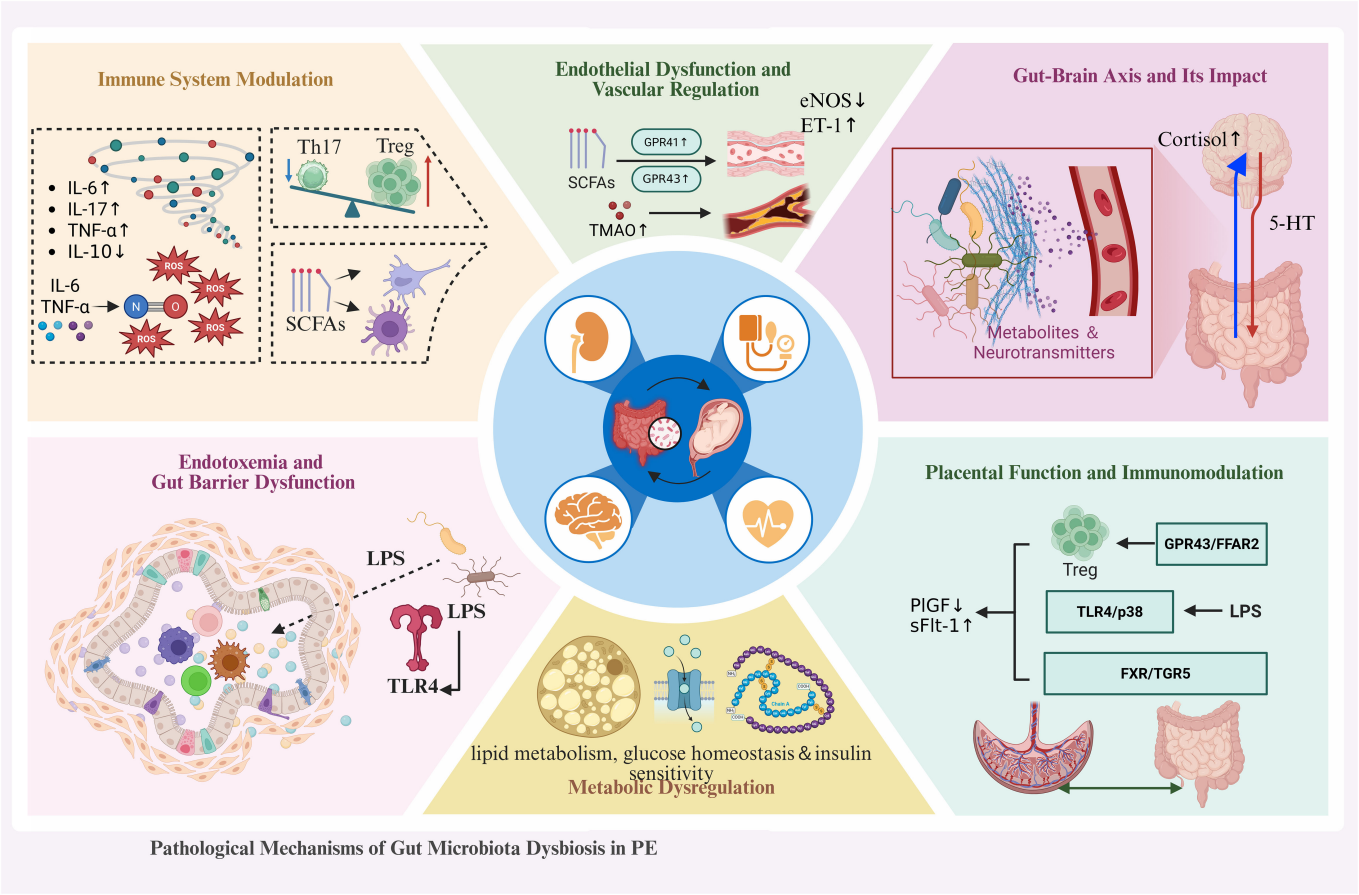
Gut microbiota dysbiosis drives PE through multi-system pathways. This schematic illustrates the novel gut-placenta axis model in PE pathogenesis. Dysbiosis triggers five interconnected pathological cascades: (1) Immune Activation - Elevated LPS activates TLR4 signaling, promoting pro-inflammatory cytokine release and Th17/Treg imbalance; (2) Metabolic Dysfunction -Reduced SCFAs impair gut barrier integrity while aberrant bile acids induce vasoconstriction; (3) Endothelial Injury – Nitric oxide reduction and ROS accumulation cause vascular dysfunction; (4) Placental Damage - Dual ischemia-inflammation insults impair trophoblast invasion and accelerate syncytiotrophoblast senescence; (5) Neuroendocrine Disruption - Gut-brain axis dysfunction deregulates RAAS, perpetuating hypertension. Key Innovation: This integrated model positions gut dysbiosis as an upstream initiator of PE, rather than merely a consequence, highlighting potential early intervention targets.

Research indicates that maintaining a balanced gut microbiota is crucial for human health (Chen et al., 2024; Isolauri and Laitinen, 2025). When the microbiome’s functions are disrupted, it can trigger a series of pathophysiological consequences. In recent years, the gut microbiota has been recognized as a critical factor influencing host health, including its involvement in pregnancy-related disorders such as PE. Dysbiosis, or the imbalance in gut microbial composition, has been shown to play a key role in the pathophysiology of PE through several molecular mechanisms (**Figure 1**). These mechanisms include immune modulation, endothelial dysfunction, gut-brain axis interactions, endotoxemia, metabolic disturbances, and alterations in placental function (Van Hul et al., 2024; Cui et al., 2025).

#### 2.2.1 Immune dysregulation: Th17/Treg imbalance and systemic inflammation

The gut microbiota shapes innate and adaptive immune responses through modulation of cytokine networks, antigen presentation, and immune cell differentiation (Belkaid and Hand Timothy, 2014; Zheng et al., 2020; Koren and Konnikova, 2024). The gut microbiota shapes innate and adaptive immune responses through modulation of cytokine networks, antigen presentation, and immune cell differentiation (Riley and Berkowitz, 2024). However, microbial dysbiosis during pregnancy disrupts this homeostasis, promoting a shift toward pro-inflammatory immunity characterized by an imbalance between pro- and anti-inflammatory cytokines (Koren et al., 2012; Münte and Hartmann, 2025).

A hallmark immune disturbance in PE is the dysregulation of the T helper 17 (Th17) to regulatory T (Treg) cell ratio. Multiple clinical studies have demonstrated that PE patients exhibit significantly elevated frequencies of Th17 cells and reduced proportions of Treg cells, accompanied by elevated circulating levels of IL-6, IL-17, and TNF-7, alongside decreased levels of the anti-inflammatory cytokine IL-10 (Darmochwal-Kolarz et al., 2012; Eghbal-Fard and Yousefi, 2019). This Th17/Treg imbalance fosters chronic systemic inflammation, which amplifies endothelial activation and vascular dysfunction-pathophysiological hallmarks of PE (Miller et al., 2022). Mechanistically, IL-6 and TNF-a promote endothelial cell dysfunction by impairing nitric oxide bioavailability, inducing oxidative stress, and upregulating adhesion molecules, thereby exacerbating placental hypoperfusion and maternal hypertension (Tenório et al., 2019; Guerby et al., 2021).

Beyond T cell-mediated immunity, gut microbiota-derived metabolites such as SCFAs modulate macrophage polarization and dendritic cell activation (Overby and Ferguson, 2021; Chen et al., 2022). A depletion of SCFA-producing taxa has been observed in PE, potentially limiting Treg cell induction and further favoring a pro-inflammatory Th17-skewed environment (You et al., 2022). This bidirectional interactional “_ENR dysbiosis exacerbates immune dysregulation while inflammation reciprocally shapes microbial composition, may form a self-reinforcing loop driving disease progression (Wan et al., 2025).

#### 2.2.2 Endothelial dysfunction and impaired vascular regulation

The gut microbiota influences endothelial function primarily through SCFAs, particularly butyrate (Maiuolo and Carresi, 2022; Luqman et al., 2024). SCFAs activate G protein-coupled receptors (GPR41 and GPR43), promoting vasodilation and endothelial integrity (Maiuolo and Carresi, 2022; Wu et al., 2024). Dysbiosis-associated reductions in SCFA production result in endothelial dysfunction and impaired vascular tone, contributing to hypertension and PE (Xue et al., 2023).

Microbial metabolites such as TMAO are also implicated in endothelial dysfunction (Wang et al., 2024; Mou et al., 2025). Elevated TMAO levels promote vascular inflammation and atherosclerosis, exacerbating hypertensive pathology (Mubeen et al., 2025), thus, gut dysbiosis converges on vascular dysfunction through multiple complementary pathways.

#### 2.2.3 Gut-brain axis disruption and neuroendocrine dysregulation

The gut microbiota communicates with the central nervous system through the gut-brain axis, influencing blood pressure regulation and stress responses (Yang et al., 2018; Kasarello et al., 2023). Gut-derived metabolites and neurotransmitters impact brain functions regulating vascular tone (Yang and Zubcevic, 2017). Dysbiosis disrupts this communication, leading to abnormal blood pressure regulationio characteristic feature of PE.

Gut-derived metabolites modulate the biosynthesis of key neurotransmitters including norepinephrine and serotonin, which regulate blood pressure and stress responses (Strandwitz, 2018; Wang and Zhao, 2018). Perturbations in the gut-brain axis, particularly microbial dysbiosis and altered neuroendocrine signaling and contribute to aberrant cardiovascular and immune regulation during pregnancy, increasing PE susceptibility (Soleimani et al., 2025; Song et al., 2025).

#### 2.2.4 Endotoxemia and intestinal barrier dysfunction

Dysbiosis increases intestinal permeability (“leaky gut”), facilitating translocation of microbial components, particularly LPS, into systemic circulation, causing metabolic endotoxemia (Cani et al., 2007; Ma et al., 2025). Circulating LPS activates TLR4, initiating systemic inflammatory cascades implicated in PE pathogenesis (Cani et al., 2021; Grylls et al., 2021). Elevated LPS enhances monocyte and macrophage activation, inducing pro-inflammatory cytokines (TNF-in IL-6, IL-1,i that aggravate endothelial dysfunction and promote hypertension (Grylls et al., 2021; Theofilis et al., 2024). Moreover, endotoxemia impairs maternal-fetal immune tolerance, disrupting trophoblast invasion and placental vascular remodeling (Tranquilli et al., 2008; Solt et al., 2025).

#### 2.2.5 Metabolic dysregulation and insulin resistance

The gut microbiota influences lipid metabolism, glucose homeostasis, and insulin sensitivity (Bäckhed et al., 2004; Qin et al., 2012; Shin et al., 2014). Dysbiosis is strongly associated with insulin resistance, lipid disorders, and impaired glucose homeostasis\l “_ENREF_9 metabolic risk factors for PE (Lin et al., 2022; Jordan et al., 2024). The microbiota modulates host energy balance through SCFA production, directly impacting glucose and lipid metabolism (Koren et al., 2012). Microbial metabolites interact with liver, adipose tissue, and skeletal muscle, influencing overall metabolic health and exacerbating PE pathophysiology (Liu et al., 2024).

#### 2.2.6 Placental dysfunction and the gut-placenta-immune axis

Maternal gut dysbiosis perturbs placental function via multiple “gut-placenta-immune” pathways (Giugliano et al., 2025). Reduced microbial diversity and expansion of opportunistic pathogens diminish SCFA production (Qing et al., 2021; Deady et al., 2024) which modulates trophoblast inflammation and promotes Treg induction via GPR43/FFAR2 receptors (Qin et al., 2025).

Dysbiosis-associated endotoxemia triggers the trophoblast TLR4/p38 axis, impairing invasion, spiral artery remodeling, and amplifying inflammation (Fan et al., 2019). Altered microbial metabolism reshapes the AHR ligand pool, with metabolites such as indole-3-lactic acid influencing trophoblast differentiation and immune mediator expression (Vinay et al.; Benech et al., 2022). Gut microbiota-driven bile acid shifts disrupt intestinal barrier integrity via FXR/TGR5 pathways, facilitating endotoxin leakage, systemic inflammation, and impaired placental angiogenesis (Larabi et al., 2023; Lin et al., 2025). These perturbations converge on anti-angiogenic imbalance, exemplified by abnormal sFlt-1/PlGF ratios (Anwer et al., 2024).

However, existing evidence remains largely correlative, with causal mechanisms obscured by confounding factors including diet, antibiotic exposure, and host genetics (Chen X. et al., 2025), Mechanistic longitudinal studies and targeted microbiome interventions with multi-omics analyses are urgently needed to clarify causality and therapeutic potential.

These six interconnected mechanisms collectively contribute to PE’s characteristic features: hypertension, proteinuria, and multi-organ dysfunction. The gut-placenta axis represents a promising frontier for novel diagnostic biomarkers and therapeutic strategies in PE prevention and management.

## 3 Placenta-derived exosomes in the pathophysiology of PE

**Figure 2:**
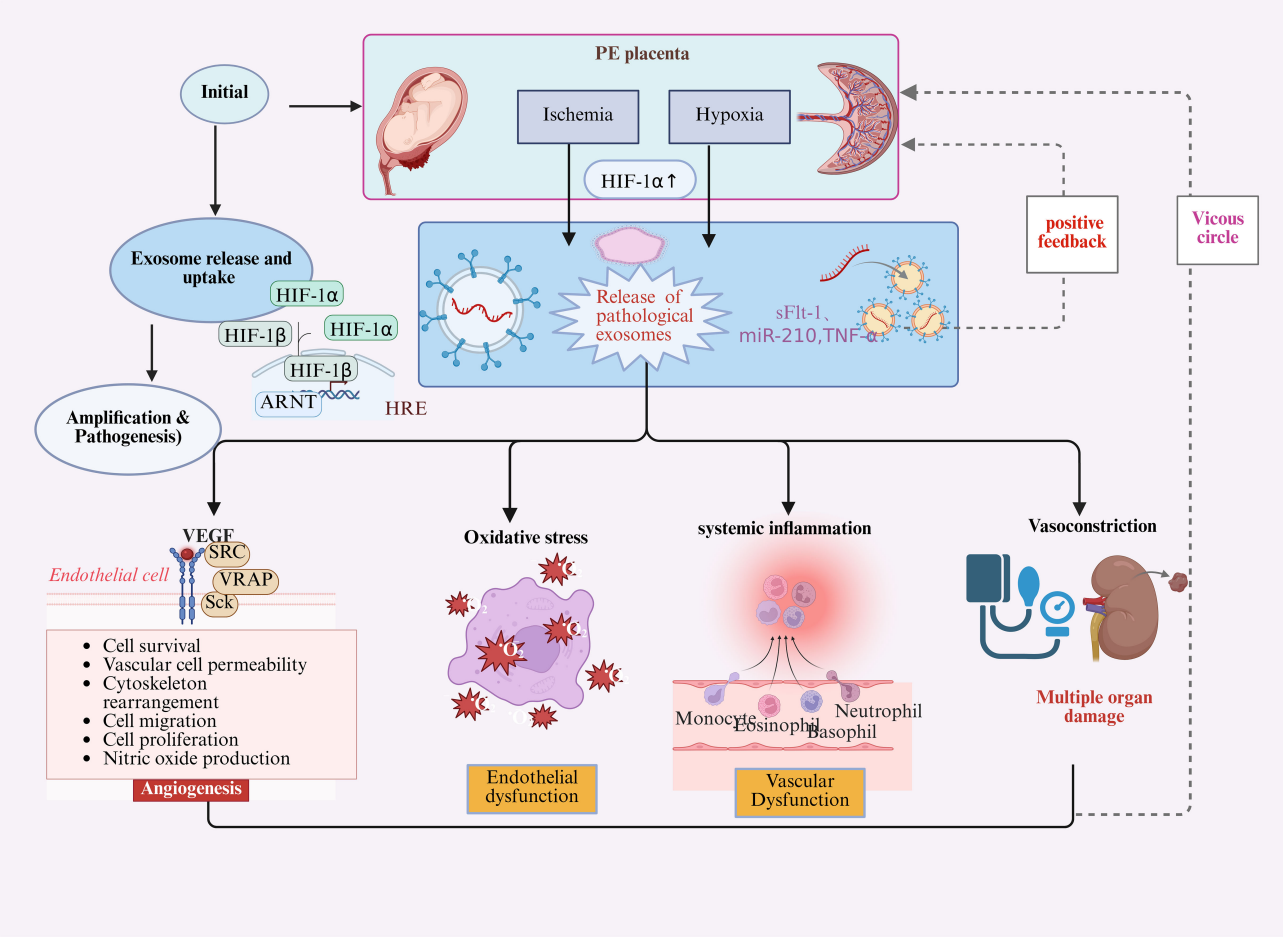
Placenta-derived exosomes mediate systemic maternal endothelial pathology in preeclampsia. Placental ischemia and hypoxia activate hypoxia-inducible factor-1i (HIF-1-1i which upregulates the biogenesis and secretion of pathological exosomes enriched with soluble fms-like tyrosine kinase-1 (sFlt-1), microRNA-210 (miR-210), and tumor necrosis factor-alpha (TNF-α). Following systemic circulation, these exosomes are internalized by maternal vascular endothelial cells and immune cells, where their molecular cargos initiate three parallel pathogenic mechanisms: (1) sFlt-1 competitively binds and sequesters vascular endothelial growth factor (VEGF) and PlGF, disrupting angiogenic balance and causing endothelial dysfunction; (2) miR-210 suppresses mitochondrial iron-sulfur cluster assembly enzymes, impairing oxidative phosphorylation and inducing reactive oxygen species production; (3) TNF-u triggers pro-inflammatory signaling cascades, activating systemic inflammation and immune cell recruitment. These mechanisms synergistically promote widespread vascular dysfunction, increased peripheral resistance, and multi-organ injury manifesting as hypertension, proteinuria, and renal/cardiovascular complications. Dashed arrows indicate positive feedback loops wherein endothelial damage amplifies placental stress and exosome secretion, establishing a self-perpetuating pathological cycle. This model positions placental exosomes as critical intercellular messengers that bridge upstream placental hypoxic stresscalslartingl initiated by gut microbiome dysbiosis in the proposed gut-placenta axisplace downstream maternal systemic pathology, offering novel mechanistic insights into preeclampsia pathogenesis and identifying exosomal components as candidate biomarkers for early detection and therapeutic intervention.

As the vital organ connecting mother and fetus, the placenta continuously releases abundant exosomes into maternal circulation. The molecular cargo and biological functions of these placental exosomes (P-Exos) undergo significant alterations during the placental explantation state, becoming key mediators of systemic maternal syndrome (Tsai et al., 2025). Recent years have witnessed growing attention on extracellular vesicles (EVs) secreted by placental trophoblast cells in placental biology and pregnancy health research (**Figure 2**). These vesicles not only play a crucial role in intercellular communication but also demonstrate significant biological functions during both normal and pathological pregnancy states.

### 3.1 Altered characteristics of placental exosomes in PE

In patients with PE, placental-derived exosomes (P-Exos) exhibit profound alterations in both quantity and molecular composition compared to normal pregnancy. Circulating exosome levels, particularly those of placental origin identified by markers such as placental alkaline phosphatase (PLAP), are significantly elevated in PE patients, likely attributable to hypoxic stress, a potent stimulus for extracellular vesicle release (Pillay et al., 2016). The pathological significance of P-Exos primarily lies in their altered cargo of bioactive molecules, which are delivered to maternal target cells to exert specific biological effects. Among protein constituents, P-Exos from PE patients are enriched with anti-angiogenic factors, most notably soluble fms-like tyrosine kinase-1 (sFlt-1) and soluble endoglin (sEng) (Palazzo et al., 2025). These exosomes contain both sFlt-1 mRNA and protein, which, upon delivery to maternal vascular endothelial cells, antagonize the pro-angiogenic actions of VEGF and PlGF, thereby representing a core mechanism underlying systemic endothelial dysfunction in PE (Huang et al., 2023)]. Additionally, P-Exos are enriched with inflammation-, coagulation-, and apoptosis-related proteins such as heat shock protein 70 (HSP70) and tissue factor, collectively exacerbating PE pathology (Adamova et al., 2022).

Regarding miRNA cargo, the most extensively studied class of P-Exos molecules, PE is characterized by substantial alterations in exosomal miRNA profiles (Chang et al., 2018). Notably, hypoxia-induced miR-210, which is upregulated in both placental tissues and circulating exosomes, can be transported to endothelial cells where it targets molecules such as Ephrin-A3, thereby inhibiting cell migration and angiogenesis and further aggravating endothelial injury (Chen Y. et al., 2025). These distinct molecular signatures, as detailed in **Table 3**, comparing placental exosome cargo between normal pregnancy and preeclampsia, underscore the critical role of P-Exos in PE pathogenesis.

**Table 3:** Placental exosome cargo: normal pregnancy vs. preeclampsia.

Cargo type	Specific examples	Change in PE	Target pathway	Functional effect
MicroRNAs	miR-155 miR-210 miR-517a/b	Upregulated Upregulated Downregulated	eNOS pathway dysfunction Angiogenesis/vascularization Trophoblast invasion	Endothelial Impaired placental Reduced invasion
Proteins	sFlt-1 sEng Placental alkaline phosphatase	Increased Increased Increased	VEGF/PlGF TGF-β signaling dysfunction Inflammatory signaling	Anti-angiogenic Endothelial Enhanced inflammation
Lipids	Ceramides	Elevated	Apoptosis, inflammation	Cell death, inflammation
mRNAs	Phosphatidylserine SERPINA3	Increased Upregulated	Coagulation Inflammatory response	Prothrombotic state Enhanced inflammation

eNOS, endothelial nitric oxide synthase.

### 3.2 Biological functions of placenta-derived exosomes

Once released into the maternal circulation, P-Exos carrying abnormal cargo act as molecular messengers, exerting widespread deleterious effects across multiple maternal systems. The diverse miRNAs and proteins contained within P-Exos can modulate signaling pathways in endothelial cells, leading to apoptosis and functional impairment. For instance, miR-144 has been shown in preeclampsia models to regulate the expression of genes associated with inflammation and endothelial function, thereby exacerbating endothelial inflammatory responses and dysfunction (Palazzo et al., 2025).

#### 3.2.1 Induction of endothelial dysfunction

Endothelial dysfunction represents a central pathophysiological mechanism in the development of PE, characterized by impaired vasodilation, hemodynamic instability, and ultimately reduced utero-placental perfusion (Matsubara et al., 2021). This compromised placental blood flow can severely restrict the transport of oxygen and nutrients to the fetus, impeding normal fetal growth and increasing the risk of intrauterine growth restriction (IUGR). Moreover, maternal cardiovascular complications such as chronic hypertension, myocardial injury, and an elevated long-term risk of cardiovascular disease are closely associated with endothelial impairment (Murugesan et al., 2022; Wu et al., 2023). Clinically, patients with PE frequently present with hypertension and proteinuria, both of which are tightly linked to endothelial damage and dysfunction.

P-Exos have emerged as important mediators in this pathological process through multiple mechanisms. First, they promote endothelial dysfunction by upregulating pro-inflammatory cytokines (e.g., IL-6, TNF-, and downregulating anti-inflammatory mediators, thereby triggering vascular inflammation and compromising the integrity of the endothelial barrier. Second, P-Exos are closely associated with oxidative stress responses, a known contributor to endothelial cell apoptosis and impaired proliferation. Reactive oxygen species (ROS) overproduction during oxidative stress can lead to cellular injury, while components within P-Exosentst as specific miRNAs and proteinststi modulate the activity of antioxidant enzymes like superoxide dismutase and catalase, influencing endothelial cell survival and functionality (Aloi and Drago, 2024). Consequently, P-Exos may exert a dual effect in PE pathogenesis, acting both as pathogenic mediators and as potential therapeutic targets for intervention.

#### 3.2.2 Modulation of maternal immune responses

During pregnancy, the maternal immune system must balance fetal tolerance and pathogen defensene, immunological paradox requiring tight regulation. Placenta-derived exosomes are emerging as critical regulators of this equilibrium by mediating immunomodulatory signaling between the placenta and maternal immune cells (Bai et al., 2021).

P-Exos are enriched with functional molecules (miRNAs, proteins, lipids, lncRNAs) that interact with T cells, B cells, NK cells, and monocytes to shape maternal immunity (Bai et al., 2022). For instance, miR-30d-5p within P-Exos promotes anti-inflammatory M2 macrophage polarization via HDAC9 suppression, aiding immune tolerance and placental repair (Shan et al., 2024).

Moreover, P-Exos influence NK cell cytotoxicity and monocyte-driven inflammation, thus maintaining immune tolerance at the maternalengn,e interface (Gebara et al., 2021). These findings underscore the dual role of P-Exos as both messengers and immune modulators in pregnancy, offering potential targets for diagnostic and therapeutic interventions (Salomon and Rice, 2017; Ander and Diamond, 2019; Hahn et al., 2019; Dauby and Flamand, 2022; Langel et al., 2022; Pogula et al., 2024).

## 4 The gut microbiota-placental exosome axis: a bidirectional regulatory network

**Figure 3:**
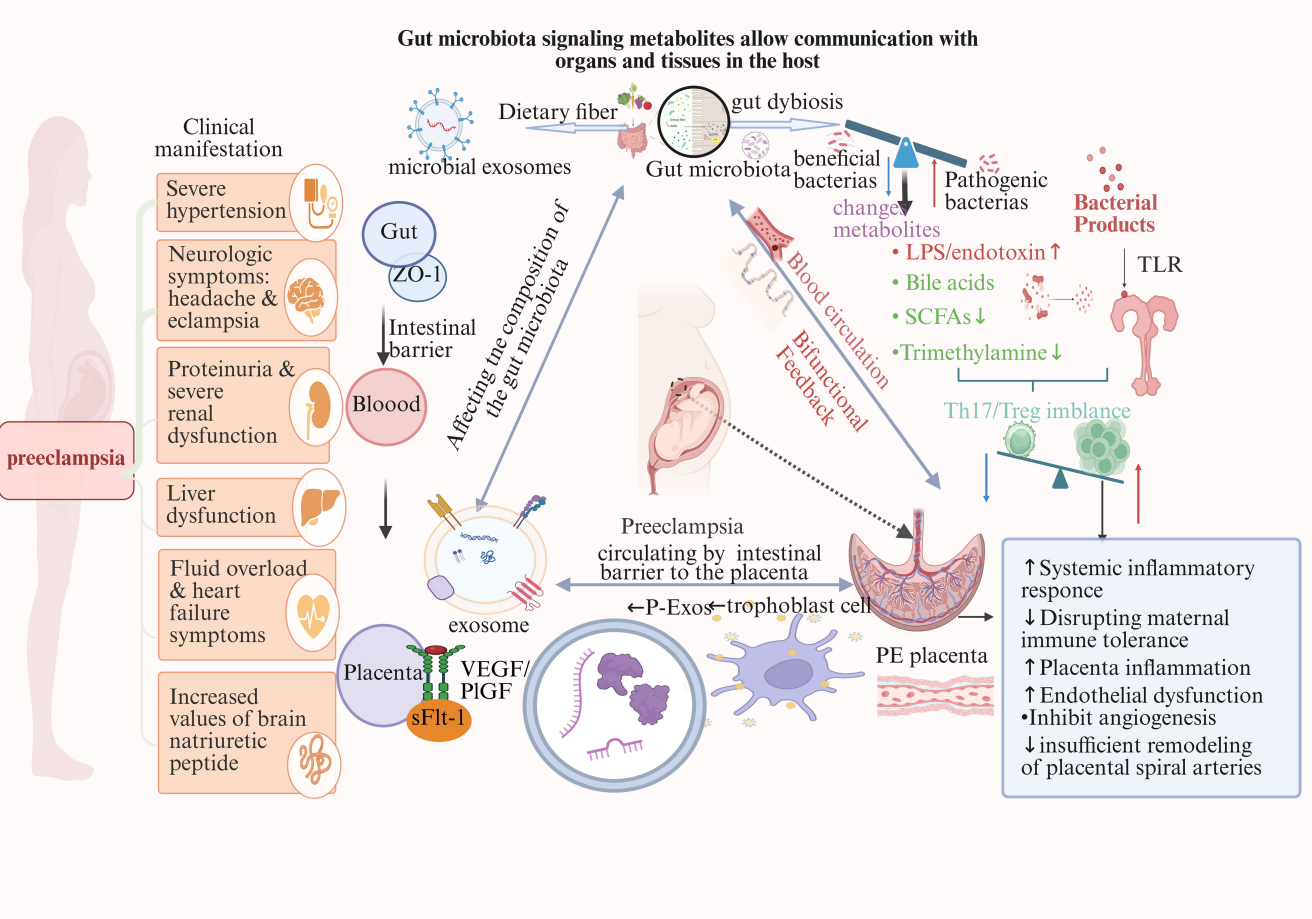
The gut microbiota-placenta-exosome axis: a novel bidirectional pathway in preeclampsia pathogenesis. This model illustrates bidirectional crosstalk between gut dysbiosis and placental dysfunction in PE. Gut dysbiosis compromises intestinal barrier integrity (reduced ZO-1), releasing LPS/endotoxin, altered bile acids, reduced SCFAs/trimethylamine, and microbial exosomes into maternal circulation. These trigger systemic Th17/Treg imbalance and immune dysregulation. Concurrently, placental hypoxia activates HIF-1at driving secretion of pathological exosomes containing miR-517a/b, miR-210, sFlt-1, and MCP-7. These exosomes amplify maternal inflammation, disrupt immune tolerance, promote endothelial dysfunction, inhibit angiogenesis, and impair spiral artery remodeling. The model reveals a self-reinforcing loop: placental inflammatory signals worsen gut dysbiosis (dashed feedback arrow), while gut-derived factors aggravate placental pathology. This bidirectional communication produces PE’s clinical manifestations: severe hypertension, proteinuria/renal dysfunction, neurological symptoms, liver dysfunction, and cardiac complications. Novelty: First comprehensive model integrating the gut-placenta-exosome axis with bidirectional feedback, identifying gut microbiota as an upstream therapeutic target and microbial/exosomal components as candidate PE biomarkers.

Emerging evidence suggests gut microbiota and placental exosomes form an integrated regulatory axis in PE pathogenesis. Groundbreaking research has demonstrated that bacterial extracellular vesicles from maternal gut microbiota can traverse biological barriers and reach amniotic fluid, potentially priming the prenatal immune system (Kaisanlahti et al., 2023). Additionally, gut dysbiosis significantly perturbs placental metabolism and immune function at the maternal-fetal interface (Giugliano et al., 2025). This section examines three key aspects of this interaction (**Figure 3**): (1) how microbial signals reach the placenta, (2) how these signals reprogram exosome cargo, and (3) how exosomes amplify microbial signals systemically.

### 4.1 Microbial signal transmission

Recent research has highlighted the gut-placenta axis as a pivotal inter-organ communication system during pregnancy (Ruiz-Triviño et al., 2023; Lu et al., 2024). Dysbiosis of the maternal gut microbiota, particularly characterized by the loss of beneficial short-chain fatty acid (SCFA)-producing bacteria (e.g., Faecalibacterium prausnitzii) and an increase in lipopolysaccharide (LPS)-producing Gram-negative bacteria, can compromise gut barrier integrity and promote systemic endotoxemia (Chen et al., 2020; Zhou et al., 2023). These circulating microbial components—collectively termed microbe-associated molecular patterns (MAMPs)—such as LPS, peptidoglycans, and bacterial DNA, may translocate across the intestinal epithelium and reach distant organs, including the placenta (Amabebe et al., 2024).

MAMPs are recognized by host pattern recognition receptors (PRRs), notably Toll-like receptors (TLRs). TLR4 activation on trophoblasts triggers NF-κB pathway activation and subsequent inflammatory cascades. In the context of PE (Fan et al., 2019), this process exacerbates systemic inflammation and disrupts immune tolerance at the maternal-fetal interface (Sun and Zhang, 2024). In parallel, the reduction of SCFAs such as butyrate impairs histone deacetylase (HDAC) inhibition and

### 4.2 Exosome reprogramming

P-Exos, small extracellular vesicles secreted predominantly by syncytiotrophoblasts, are emerging as potent mediators of maternal-fetal communication. Microbial signals, whether endotoxins like LPS or metabolites such as SCFAs—can influence both the biogenesis and cargo packaging of these vesicles.

On one hand, LPS exposure enhances P-Exos secretion via TLR4-mediated activation of the NF-κB and MAPK pathways, increasing the exosomal load of pro-inflammatory molecules, including miR-210, miR-155, and IL-6 (Zhan et al., 2022; Meng et al., 2023). On the other hand, benefit miRNAs es like butyrate may exert protecregulations by promoting the packaging of anti-inflammatory miRNAs (e.g., miR-146a) through epigenetic regulation (Parada Venegas et al., 2019; Siddiqui and Cresci, 2021).

Thus, the microbial environment decisively influences whether P-Exos convey “protective” or “pathological” messages. A proposed model suggests: gut dysbiosis leads to increased systemic LPS and decreased butyrate levels, which in turn activates TLR4/NF-κB in trophoblasts (Zhang et al., 2025). This activation results in the loading of pathological miRNA/mRNA cargo into exosomes, leading to the release of “PE-primed” P-Exos into the maternal circulation (Kaur et al., 2025). This, in turn, further impairs endothelial and immune homeostasis.

### 4.3 Systemic signal amplification

Emerging evidence demonstrates that alterations in the maternal gut microbiota composition and metabolic functions are closely linked to maternal health during pregnancy, with microbial-derived SCFAs playing pivotal roles in modulating the maternal immune-metabolic milieu (Wu et al., 2022). Recent studies reveal that maternal SCFAs traverse the placenta, influencing fetal development via placental transport mechanisms (Qin et al., 2025). Simultaneously, placenta-derived extracellular vesicles (P-Exos), including exosomes, serve as biological amplifiers of such low-level microbial signals by facilitating their systemic dissemination (Rosenfeld, 2024). For instance, SCFAs not only exert anti-inflammatory effects in the maternal gut but may also be packaged within P-EXOs and conveyed to the placenta, where they regulate placental growth and function (Rosenfeld, 2024). Furthermore, P-Exos critically regulate maternal systemic immune responses: they reprogram circulating monocytes toward an immunosuppressive, M2−polarized profile and promote expansion of regulatory T cells, reinforcing maternal–fetal immune tolerance essential for successful pregnancy (Bai et al., 2022). In pathological states such as preeclampsia (PE), where maternal immune tolerance is compromised, P-Exos exhibit altered cargo with potential to modulate immune homeostasis and mitigate disease pathology (David and Maharaj, 2025). The intrinsic capacity of P-Exos to “package” and “concentrate” signaling molecules enhances their stability and bio-activity, enabling precise modulation across varying physiological and pathological settings (Bai et al., 2021). This mechanism not only provides a novel lens through which to understand the gut microbiota–P-Exos axis in pregnancy-related disorders, including PE, but also underscores the therapeutic potential of modulating maternal gut microbiota to influence P-Exos function for the prevention and treatment of pre-eclampsia.

## 5 Exos impact on the maternal-fetal immune micro-environment and vascular function

### 5.1 Remodeling the maternal-fetal immune micro-environment

P-Exos, “programmed” by gut microbiota, ultimately drive the clinical phenotype of PE by modulating maternal immune and vascular systems (Giugliano et al., 2025). A successful pregnancy depends on finely tuned immune tolerance at the maternal–fetal interface, which is disrupted in PE, shifting toward a pro-inflammatory state. Placenta-derived exosomes enriched with pro-inflammatory molecules such as miR−155 play a pivotal role in this process. Upon uptake by circulating maternal monocytes, these exosomes promote differentiation into M1-like pro-inflammatory macrophages that secrete elevated levels of TNF-α and IL-6 (Shen et al., 2018).

Concurrently, P-Exos impair the suppressive function of regulatory T (Treg) cells while promoting expansion and activation of Th1 and Th17 subsets, leading to an imbalance in the cytokines milieu (He et al., 2025). This systemic immune activation exacerbates endothelial injury and may further disrupt the decidual immune microenvironment, impairing placental function and propagating a vicious cycle.

### 5.2 Disruption of maternal vascular function

P-Exos are key conveyors of anti-angiogenic signals. Exosomes carrying sFlt-1 and miR−210 fuse with maternal endothelial cells, directly impairing their function. sFlt-1 acts by sequestering VEGF and PlGF, neutralizing their pro-angiogenic activity, while miR−210 inhibits endothelial repair and migration. The combined effects include: (1) Decreased eNOS activity and reduced nitric oxide (NO) production, leading to impaired vasodilation and elevated blood pressure (Shen et al., 2018); (2) Compromised e) dothelial junction integrity, resulting in incr;(s)d vascular permeability, proteinuria, and edema; (3) Upregulated procoagulant molecules and downregulated anticoagulant factors on endothelial surface, creating a hypercoagulable state; These P−Exos mediated vascular injuries effectively explain the main clinical features of PE.

Importantly, maternal environmental factors—such as a high-fat diet—can predispose to vascular dysfunction. Such diets have been shown to increase offspring blood pressure and impair maternal aortic endothelial homeostasis, associated with elevated ROS and pro-inflammatory cytokines, e.g., IL−1β, TNF−α, IL−6 (Shi et al., 2024). Inhibition of NLRP3 or IL-1β has been demonstrated to ameliorate these vascular impairments and reduce inflammation in models exposed to high-fat diets.

## 6 Clinical implications and future perspectives

**Figure 4:**
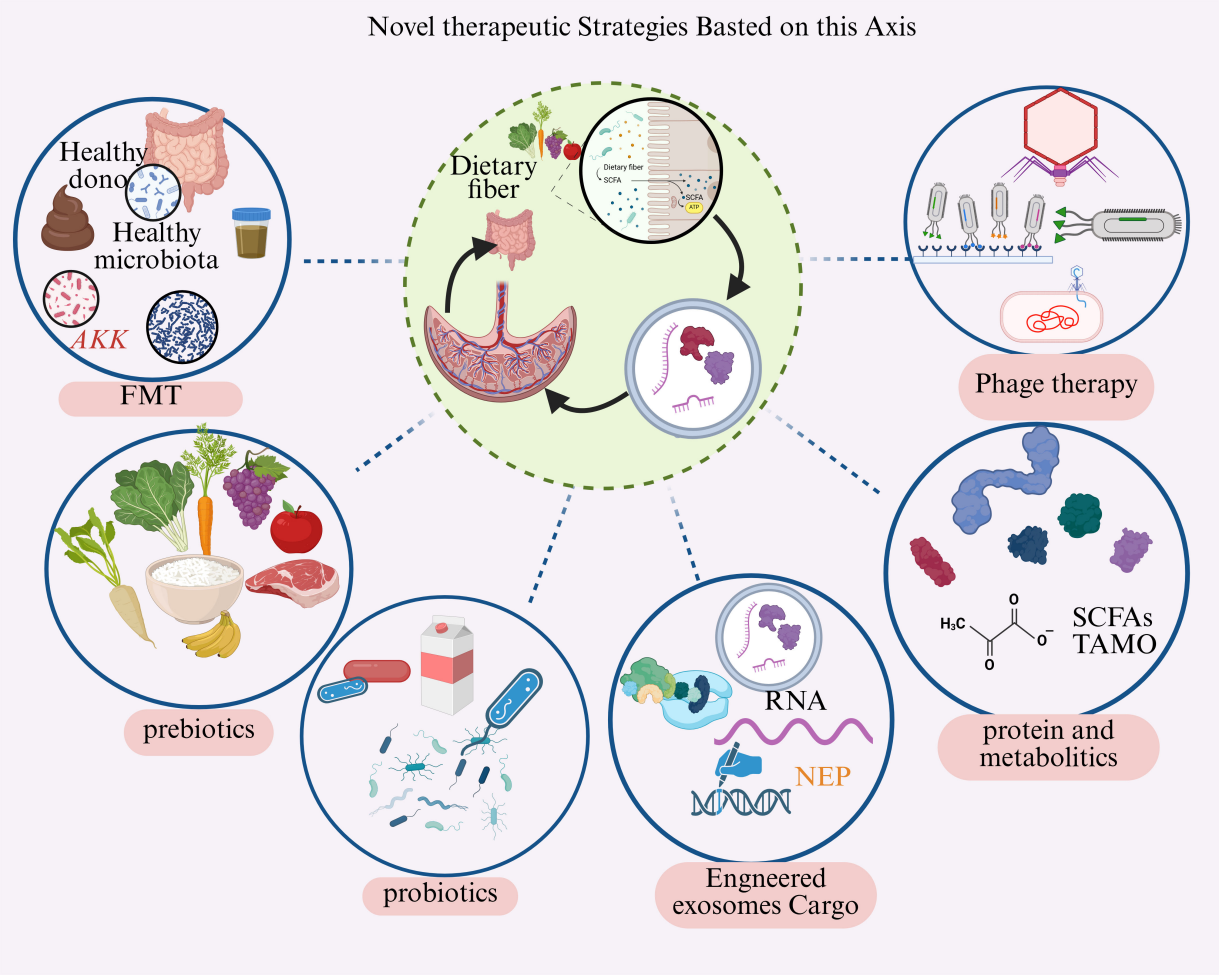
Novel microbiome-targeted therapeutic strategies for preeclampsia prevention and management. This schematic presents a comprehensive therapeutic framework targeting the gut microbiota-placenta-exosome axis for PE intervention. The central green circle illustrates the bidirectional gut-placenta crosstalk established in our model, which serves as the mechanistic foundation for six complementary therapeutic approaches: (1) FMT from healthy donors to restore eubiosis and break the pathological cycle; (2) Prebiotics (functional carbohydrates, dietary fiber) to selectively promote beneficial microbial growth; (3) Probiotics (live beneficial bacteria such as Lactobacillus and Bifidobacterium) to directly replenish protective strains; (4) Phage therapy using bacteriophages to selectively eliminate pathogenic bacteria while preserving commensal flora; (5) Engineered exosome cargo modified with therapeutic RNA or neuroprotective peptides (NEP) to counteract pathological exosome effects; (6) Direct supplementation of beneficial metabolites including short-chain fatty acids (SCFAs) and trimethylamine N-oxide (TAMO) modulators to bypass dysbiotic metabolic deficiencies. Novelty: This is the first integrated therapeutic strategy specifically designed to target multiple nodes of the gut-placenta-exosome axis simultaneously, offering a paradigm shift from symptomatic management to mechanistic intervention. By addressing the upstream gut dysbiosis and intercellular exosome communication pathways identified in our model, these approaches provide rational, personalized prevention and treatment options for PE with potential applicability before clinical symptom onset.

A comprehensive understanding of the gut microbiota-placental exosome (P-Exo) axis provides not only novel mechanistic insights into the pathogenesis of pre-eclampsia (PE) but also opens new avenues for early diagnosis, risk prediction, and targeted interventions (**Figure 4**). Current PE diagnosis is largely symptom-based after 20 weeks of gestation, when therapeutic opportunities are limited (Salomon et al., 2017). The gut microbiota, P-Exo axis offers a rich source of candidate biomarkers, including established indicators such as reduced plasma placental acid, the factor PlGF and elevated maternal serum dysfunction (both linked to hypertension and placental dysfunction (Almaani, 2019; Corominas et al., 2022).

In addition, disease-specific long noncoding RNAs (lncRNAs) identified within exosomes from PE placentas, as well as metabolite signatures measured via LC-MS/MS, show promise for stratifying high-risk pregnancies (Su et al., 2021). Alterations in gut and vaginal microbiota, through effects on immune modulation and inflammation, are also emerging as predictive markers (Valiati et al., 2023). Importantly, exosomal profiling qualifies as a “liquid biopsy” approach non-invasive, repeatable, and compatible with high-sensitivity detection platforms such as digital PCR and microfluidic exosome chips—facilitating ultra-early PE screening (Li et al., 2023).

Therapeutically, modulating the gut microbiota has emerged as a viable strategy to influence the P-Exo axis. Probiotics, particularly Lactobacillus and Bifidobacterium species, restore microbial balance, enhance SCFA production, and strengthen gut barrier integrity, thereby reducing systemic inflammation (Li et al., 2022),Clinical data suggest that probiotic intake during pregnancy may be associated with reduced risk of adverse outcomes, including gestational diabetes and preterm birth (Nordqvist et al., 2018), Prebiotics—dietary fibers that promote beneficial bacterial growth, further support SCFA synthesis and microbiota diversity, with potential benefits for both maternal health and fetal development. FMT, though not yet has demonstrated efficacy in restoring gut microbial equilibrium in dysbiosis-related diseases and may hold future promise (Jia et al., 2025). Precision dietary interventions, such as increasing fiber intake to elevate SCFA production or reducing choline/L-carnitine intake to lower TMAO levels, offer safe, adjunctive means to modulate vascular and immune pathways in pregnancy (Than et al., 2022).

Direct targeting of placental exosomes presents another therapeutic frontier. As **Table 4**, Potential strategies include: (1) inhibiting pathological exosome release by modulating regulatory molecules such as CREG1, which promotes trophoblast-derived exosome biogenesis via IGF2R signaling; (2) silencing HIF-1α to enhance trophoblast invasion and ameliorate PE pathology; (3) clearing circulating pathogenic exosomes—particularly those enriched in anti-angiogenic factors like sFlt-1, through extracorporeal filtration devices analogous to hemodialysis; (4) blocking P-Exo uptake by endothelial cells using small molecules or monoclonal antibodies that interfere with receptor-mediated endocytosis; and (5) engineering therapeutic exosomes loaded with protective miRNAs (e.g., miR-21) or pro-angiogenic factors, which have been shown in PE animal models to restore endothelial function and promote placental vascularization, Despite recent progress, significant gaps remain. Most studies to date are observational, precluding firm conclusions about causality —whether gut dysbiosis initiates PE or whether PE pathology alters microbiota composition remains unclear. The molecular mechanisms by which microbial signals influence exosome cargo sorting-potentially involving ESCRT complexes, ALIX, and epigenetic modifications—are incompletely understood (Gál et al., 2024).Other microbial niches, including oral, vaginal, and placental microbiomes, likely interact with gut flora and P-Exos in PE pathophysiology, representing an underexplored dimension of integrated microbiome research. Furthermore, existing animal models have limited translational relevance due to differences in placental architecture and immune regulation; advanced human-relevant systems, such as placental perfusion models and trophoblast organoids, are urgently needed (MacDonald et al., 2022). Future research priorities include establishing large, multicenter prospective cohorts beginning in early pregnancy or preconception; applying single-cell sequencing and spatial transcriptomics to map P-Exo signaling at cellular resolution; and employing gene-edited and germ-free animal models to validate the roles of specific microbial strains and metabolites in P-Exo-mediated PE pathogenesis.

**Table 4:** Therapeutic strategies targeting the gut-placenta axis in preeclampsia.

Intervention	Mechanism	Study status	Key findings	Limitations
Probiotics (Lactobacillus, Bifidobacterium)	Microbiota restoration, SCFA enhancement, immune modulation	Clinical trials(Phase II/III)	Mixed results; some showed BP reduction in at-risk women	Optimal strain, dose, timing unclear
Prebiotics (inulin, FOS)	Selective growth of beneficial bacteria	Preclinical +pilot studies	Enhanced SCFA production, improved gut barrier	Limited human data in pregnancy
Postbiotics (butyrate, propionate)	Direct SCFA supplementation, anti-inflammatory	Early preclinical	Bypasses microbiota variability; promising in animal models	Dosing, safety in pregnancy unknown
FMT	Comprehensive microbiota remodeling	Early preclinical (animal models) PE in rat models	Dramatic microbiota restructuring; prevented timing critical	safety concerns, ethical issues,
MSC-derived exosomes	Anti-inflammatory exosome delivery, cargo modification	Preclinical	Reduced placental oxidative stress and inflammation in models	Production, standardization challenges
Dietary interventions	Whole-system approach, fiber ↑ processed foods ↓	Observational studies	Mediterranean diet associated with lower PE risk	Compliance, confounders
Exosome inhibitors	Block pathogenic exosome uptake or biogenesis	Early preclinical (*in vitro*)	Reduced endothelial dysfunction in culture	Specificity, delivery to placenta

Collectively, elucidating the bidirectional interactions between gut microbiota and placental exosomes will be pivotal for developing standardized diagnostic platforms and precision therapeutic strategies, ultimately improving maternal and fetal outcomes in preeclampsia.

## 7 Conclusion

The emerging gut-microbiota-placental-exosome axis represents a paradigm shift in preeclampsia research, revealing previously unrecognized mechanistic links between maternal microbiome homeostasis and placental dysfunction (Le Bras, 2023). This review synthesizes evidence for three key conceptual advances that fundamentally broaden our understanding of PE pathogenesis:

First, PE pathogenesis extends beyond local placental factors to encompass systemic, microbiota-driven processes. Gut dysbiosis, characterized by reduced beneficial bacteria (Lactobacillus, Bifidobacterium) and increased pathobionts (Proteobacteria)-disrupts production of protective metabolites, particularly SCFAs (Torres-Torres et al., 2025), while increasing systemic exposure to pro-inflammatory bacterial products such as LPS (Qing et al., 2021). These alterations directly modulate placental function through multiple pathways including immune dysregulation, endothelial damage, and oxidative stress.

Second, placental exosomes function as critical mediators of maternal-fetal communication, with cargo composition fundamentally altered in PE. Increased levels of anti-angiogenic proteins (sFlt-1, sEng), pro-inflammatory microRNAs (miR-155, miR-210), and lipotoxic molecules (ceramides) packaged within P-Exos drive the systemic endothelial dysfunction and immune activation characteristic of PE. Importantly, this exosomal cargo reflects and amplifies gut dysbiosis signals, creating a feed-forward pathogenic loop.

Third, this axis operates bidirectionally. Not only does gut dysbiosis influence placental exosome production and cargo, but placental stress signals may reciprocally alter maternal gut microbiota composition through systemic inflammatory mediators and altered metabolic demands (Li et al., 2025). This bidirectional communication establishes PE as a systems-level disorder requiring integrated therapeutic approaches.

These insights illuminate transformative opportunities for clinical translation of the gut-placenta-exosome axis paradigm in preeclampsia management. Integration of gut microbiota signatures (via 16S rRNA sequencing or targeted metabolomics) with circulating exosomal miRNA profiles holds promise for developing composite biomarkers capable of predicting PE onset during early pregnancy, preceding clinical manifestation (Cao et al., 2024). Furthermore, microbiota modulation through evidence-based probiotic, prebiotic, or dietar interventions administered during the periconception period or early gestation represents a viable primary prevention strategy, particularly for high-risk populations. Novel therapeutic avenues targeting placental exosome biogenesis, cargo modification, or blockade of pathogenic exosome uptake by maternal endothelium remain largely unexploited and warrant systematic investigation.

However, critical knowledge gaps must be addressed to advance this field. Establishing causal relationships between microbiota dysbiosis and PE pathogenesis through rigorously designed interventional studies remains paramount. The molecular mechanisms governing exosome uptake, intracellular trafficking, and functional cargo delivery in target cells require comprehensive elucidation. Optimal parameters for microbiota-based interventions—including timing, composition, dosage, and delivery modalities—demand systematic determination. Additionally, integrating multi-omic platforms (metagenomics, metabolomics, proteomics, and exosome profiling) will enable refined patient stratification and personalized intervention strategies. Importantly, validation across ethnically diverse populations and varied pregnancy contexts is essential to ensure generalizability.

The gut-placenta-exosome axis framework provides a systems-level foundation for developing precision medicine approaches to PE. Translating these mechanistic insights into evidence-based clinical interventions through multicenter trials could substantially mitigate the global burden of this life-threatening pregnancy complication.
